# Beyond Participation: Adolescents’ Perspectives on Meaningful Engagement and Data Quality in Health Research

**DOI:** 10.1192/j.eurpsy.2025.840

**Published:** 2025-08-26

**Authors:** M. Karimipour, E. Soneson, M. Fazel

**Affiliations:** 1 Psychiatry, University of Oxford, Oxford, United Kingdom

## Abstract

**Introduction:**

A lack of participation from representative populations, limited long-term engagement, and potentially inaccurate self-reported responses have compromised the validity of health research findings in adolescents. Despite positive trends in some areas of adolescent health, others—such as mental health—have alarmingly deteriorated, highlighting the continuous need for adolescent health research. Therefore, it is vital to explore barriers to meaningful participation of adolescents and the factors that contribute to decreased data quality.

**Objectives:**

This study aimed to examine adolescents’ preferences and concerns about participating in health research to inform strategies for improving their likelihood of participation, meaningful engagement, and the provision of accurate responses.

**Methods:**

Nine focus groups were conducted at three secondary schools and two sixth form colleges in England to explore the perspectives of 46 adolescents aged 16 to 18 years. The constructivist grounded theory methodology was employed to develop a framework that offers insights into the mechanisms influencing young people’s participation, engagement, and provision of accurate responses in health research.

**Results:**

“Five categories emerged as underlying factors that contribute to adolescents’ participation, meaningful engagement, and provision of accurate responses. Participants suggested that a (1) *Positive Relationship with Researchers* cultivates an atmosphere of (2) *Emotional Security*, which in turn minimises the potential influence of (3) *Others’ Judgments.* If adolescents are given some (4) *Choice and Control* over research processes and the data they provide, they are more likely to feel secure, which encourages continued engagement and accurate responses. The combination of a *Positive Relationship with Researchers* and young people’s *Choice and Control* over research processes allows them to perceive their participation as an (5) *Impactful Collaboration in Research Processes*, empowering them to participate and provide accurate answers, especially to questions about their personal life experiences.”

**Image 1:**

**Figure:**
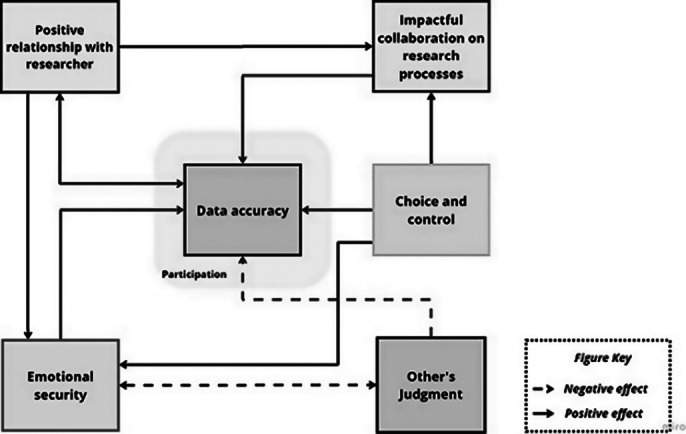

**Image 2:**

**Figure:**
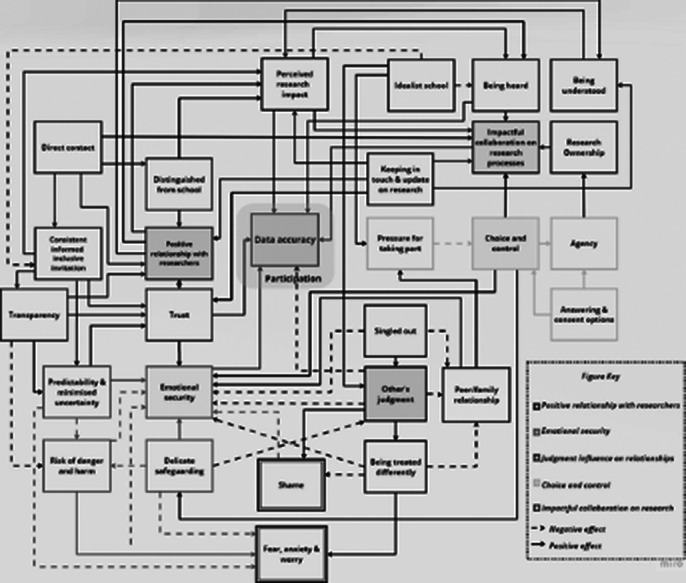

**Conclusions:**

This study suggests that adolescent health research would improve by investing more in building positive relationships with young people. This can be achieved by offering them choice and control over study processes as much as feasible, as well as by highlighting the expected impacts of their participation. Given the concerns surrounding judgment and potential legal or safeguarding issues related to research participation, open and reassuring discussions about these aspects would support adolescents’ emotional security throughout the research process. The current study recommends balancing attention between research processes and the emotional and relational aspects of adolescents’ experiences of health research.

**Disclosure of Interest:**

None Declared

